# Inhibition of VEGF_165_-Induced Angiogenesis by Gold Nanoparticles through HQGQH as the Primary Binding Site

**DOI:** 10.1021/acs.jcim.5c02810

**Published:** 2026-04-17

**Authors:** Jiayi Wan, Yihan Zhou, Jiajun Zhu, Yutong Lu, Yuting Lyu, Hongke Hao, Zhiliang Lu, Carrie A. Duckworth, Yiheng Liu, Hanyu Zhou, Yulan Yi, Kevin Chun Chan, Xia Huang

**Affiliations:** † Department of Biosciences and Bioinformatics, Xi’an Jiaotong-Liverpool University, Suzhou, Jiangsu 215123, P. R. China; ‡ Institute of Systems, Molecular and Integrative Biology, The University of Liverpool, Biosciences Building, Crown Street, Liverpool L69 7BE, U.K.

## Abstract

Inhibition of vascular endothelial growth factor 165 (VEGF_165_)-mediated angiogenesis is a promising anticancer strategy. While gold nanoparticles (AuNPs) are effective inhibitors of VEGF_165_, the precise molecular interface of their interactions with VEGF_165_ is unclear, hindering rational therapeutic design. This study combined molecular dynamics (MD) simulations and experimental techniques to map the binding interface between AuNPs and VEGF_165_. We discovered that AuNPs preferentially bind to the HQGQH motif on VEGF_165_. Functional assays showed that this binding inhibited VEGF_165_-induced endothelial cell migration and angiogenesis. The necessity of the HQGQH motif was unequivocally demonstrated by mutagenesis. A 5A-substituted VEGF_165_ mutant exhibited markedly reduced AuNP binding affinity across binding free energy calculations, ELISA, and ζ potential measurements, and its angiogenic activity became resistant to AuNP inhibition to some extent. These findings reveal the molecular basis of AuNP-mediated VEGF_165_ blockade, opening avenues for designing targeted antiangiogenic nanomedicines.

## Graphical abstract

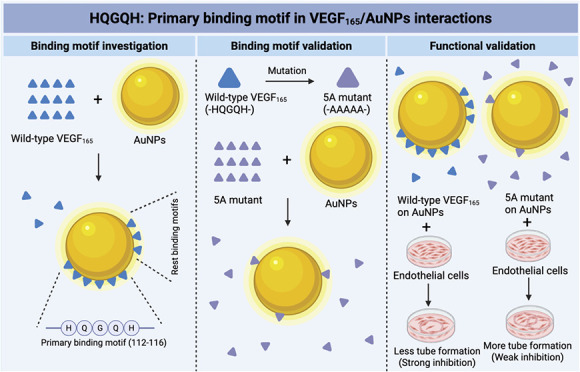

## Introduction

Tumor angiogenesis is a critical biological process where new blood vessels sprout from pre-existing vasculature to supply oxygen and nutrients to the growing tumor mass.^1,2^ This process is indispensable for tumor survival and plays a crucial role in promoting tumor growth, invasion, and metastasis.^3,4^ Angiogenesis is tightly regulated by the dynamic interplay between pro-angiogenic and antiangiogenic factors, with tumors often shifting this balance toward pro-angiogenic factors to support tumor vascular growth.^5,6^ Vascular endothelial growth factor (VEGF) is a key pro-angiogenic factor of tumor angiogenesis. VEGF-A, the most extensively studied isoform, is predominantly secreted by tumor cells and tumor-associated stromal cells.^7^ Upon binding to VEGF receptor 2 (VEGFR-2), VEGF-A activates intracellular signaling cascades that promote endothelial cell proliferation and migration, ultimately leading to the formation of aberrant vascular network within the tumor microenvironment.^8^ VEGF_165_ is a functionally significant subtype among various isoforms of VEGF-A. It is a homodimer composed of a heparin-binding domain (HBD) and a receptor-binding domain (RBD).^9^ The RBD of VEGF_165_ exhibits high-affinity interactions with VEGFR-2 and facilitates angiogenic responses to promote vascularization.^10^ Antiangiogenic therapies have demonstrated significant tumor-suppressive effects by targeting key regulatory pathways involved in tumor vascularization. These therapeutic strategies function by inhibiting VEGF-dependent angiogenic signaling, thereby disrupting the formation of tumor vasculature and impeding tumor progression. For example, bevacizumab is a recombinant humanized monoclonal antibody with Food and Drug Administration approval, whose mechanism of action is to bind to and neutralize VEGF, thereby inhibiting tumor vessel growth. Bevacizumab inhibits angiogenesis by specifically binding to the RBD of VEGF-A, thereby preventing its interaction with VEGFR-2 and directly neutralizing VEGF signaling.^11^ Bevacizumab has been extensively used in antiangiogenetic therapies for the treatment of colorectal cancer, breast cancer, nonsmall-cell lung cancer, glioblastoma, renal cell carcinoma, ovarian cancer and cervical cancer.^12^

Nanoparticles have also emerged as promising agents for inhibiting tumor angiogenesis, as they can directly adsorb and bind pro-angiogenic factors to form a protein corona, thereby disrupting pro-angiogenesis signaling. Nanoparticles are nano-objects ranging from 1 to 100 nm.^13^ AuNPs are inorganic nanoparticles that have potential in cancer diagnosis and therapy. Their unique optical properties make them suitable for tumor visualization and bioimaging, such as magnetic resonance imaging, photoacoustic imaging, and X-ray scatter imaging.^14^ Additionally, due to localized surface plasmon resonance effects, AuNPs have been used in photothermal therapy of cancer by inducing cancer cell death by heat.^15^ Moreover, the high surface-to-volume ratio of AuNPs allows high loading amounts, where AuNPs can conjugate and interact with different molecules, including drugs, nucleic acids, and proteins.^16,17^ For instance, AuNPs could directly bind to VEGF_165_ at the HBD and block VEGF_165_-induced signaling.^18^ Such blocking led to the disruption of the binding between VEGF_165_ and VEGFR-2 and inhibited the phosphorylation of VEGFR-2 and subsequent intracellular signaling events in a size-dependent manner.^19^ Consequently, AuNPs inhibited VEGF_165_-induced endothelial cell proliferation, migration, and tube formation through the Akt signaling pathway.^20^ In the tumor microenvironment, AuNPs could also bind to VEGF_165_ secreted from cancer cells, cancer-associated fibroblasts, and endothelial cells and disrupt the signal transduction from these cells to endothelial cells, thus inhibiting angiogenic phenotypes via blockade of VEGF–VEGFR-2 signaling *in vitro*.^21^ Furthermore, since the folic acid receptor is highly expressed in many types of cancers, researchers applied polymer and folic acid-modified gold nanoparticles (AuNPP-FA) to abnormal tumor vessels and found AuNPP-FA inhibited tumor angiogenesis and controlled tumor metastasis by inhibiting endothelial Smad2/3 signaling *in vivo*.^22^ Beyond AuNPs, other nanomaterials such as nanodiamonds have also shown antiangiogenic effects, reducing tumor vascular proliferation in mouse models.^23^

Although the inhibitory effect of AuNPs on VEGF_165_-mediated angiogenesis has been established, relatively few studies reported the specific amino acid residues responsible for mediating VEGF_165_/AuNPs interactions. Previous research introduced a single point mutation in peptide fragment VEGF_73–101_ by replacing G88 with C88 and observed changed ζ potential values for VEGF/AuNPs, highlighting the crucial contribution of G88 in VEGF/AuNPs interactions.^24^ However, the precise binding motif involved in this process remains unclear. Identifying the key binding motif of VEGF_165_ in VEGF_165_/AuNPs interactions is important to reveal the molecular mechanism of this process and has the potential to provide novel and precise therapeutics for the development of antitumor treatments. MD simulation is potential to explore VEGF_165_/AuNPs interactions. MD simulation is a fundamental methodology of molecular modeling and computational design that is used to investigate the temporal evolution and dynamics of molecular systems.^25^ MD simulations enable atomistic-resolution tracking of biomolecular processes such as conformational changes, ligand binding, and protein folding at femtosecond time scales, and also predict molecular responses to perturbations, including mutations, phosphorylation, and protonation.^26^ Recently, such simulations have also been used to explore the interactions between proteins and nanoparticles or drugs in nanomedicine. For example, researchers used coarse-grained MD simulations of AuNPs in interactions with insulin and fibrinogen to explore the binding modes, protein structure changes, and binding sites.^27^ Similarly, researchers used the all-atom MD to investigate the mechanism of molecular recognition between Macugen and VEGF_165_, and identified residues Cys10, Ser11, Lys26, and Cys27 from the HBD of VEGF_165_ in the interacting interface.^28^ Another study applied molecular docking, MD methodologies, and circular dichroism spectroscopy to model the whole structure of VEGF and to provide potential molecular mechanisms governing the VEGF/VEGF receptor/heparin system based on free energy analysis.^29^ Steered molecular dynamics (SMD) simulation uses a time-dependent external force to drive the system to move in a predefined way.^30^ It has emerged as a powerful tool to qualitatively estimate binding affinities through nonequilibrium work measurements.^31^ In addition, umbrella sampling (US) is a widely used method for computing the PMF of noncovalent ligand–receptor dissociation processes identified in SMD, providing more thermodynamic details.^32^

In this study, we employed MD simulations to establish an VEGF_165_/AuNPs interaction model and identified the binding motif in this process. The identified binding site was replaced by five alanine residues to determine its functional significance in the interaction. We compared the binding energy values between AuNPs and either wild-type VEGF_165_ (WT) or the 5A mutated VEGF_165_ (5A mutant). This comparative analysis was further applied in ELISA for binding rate evaluation and ζ potential measurements for surface charge assessment. Moreover, migration and angiogenesis assays were conducted to examine whether disruption of this binding mode affects VEGF_165_-mediated migration and angiogenesis, thereby confirming the biological importance of this interaction at the cellular level. Here, we aim to identify and characterize the essential amino acid residues within VEGF_165_ responsible for binding to AuNPs. This work provides new insights into the mechanism underlying the VEGF_165_/AuNPs interaction and contributes to the development of potential antiangiogenic therapies in cancer treatment.

## Results

### The Conformational Dynamics of VEGF_165_

A comprehensive understanding of the conformational dynamics of VEGF_165_ under physiological conditions is crucial for elucidating its potential binding interface with nanoparticles and achieving effective inhibitory effects. However, previous molecular dynamics studies on VEGF_165_ were largely limited to short time scales,^29^ which proved insufficient to capture the full structural landscape of the protein. To overcome these limitations and obtain deeper insights into VEGF_165_’s intrinsic conformational dynamics and its implications for nanoparticle interactions, we conducted four independent 2000 ns MD simulations. Principal Component Analysis (PCA) of the heavy atom coordinates from these trajectories (Supporting Figure S1) revealed two predominant conformational states of a VEGF_165_ dimer: an extended state and a compact state (Figure 1A). The extended state is characterized by spatially separated and more flexible dangling regions with higher solvent accessibility, while the compact state features close proximity of the two dangling regions stabilized by interchain hydrogen bonds and reduced solvent-accessible surface area. To quantitatively characterize these observed conformational changes, the RMSD of the dangling region was calculated for each frame after alignment to the final structure (Figure 1B). The RMSD displayed a consistent decreasing trend throughout the 2000 ns simulations, indicating a progressive conformational stabilization toward its compact state. Consistently, the distance between the COMs of the two dangling regions in VEGF_165_ dimer showed a clear decrease over time (Figure 1C), further supporting the gradual approach and compaction of these regions.

**Figure 1:**
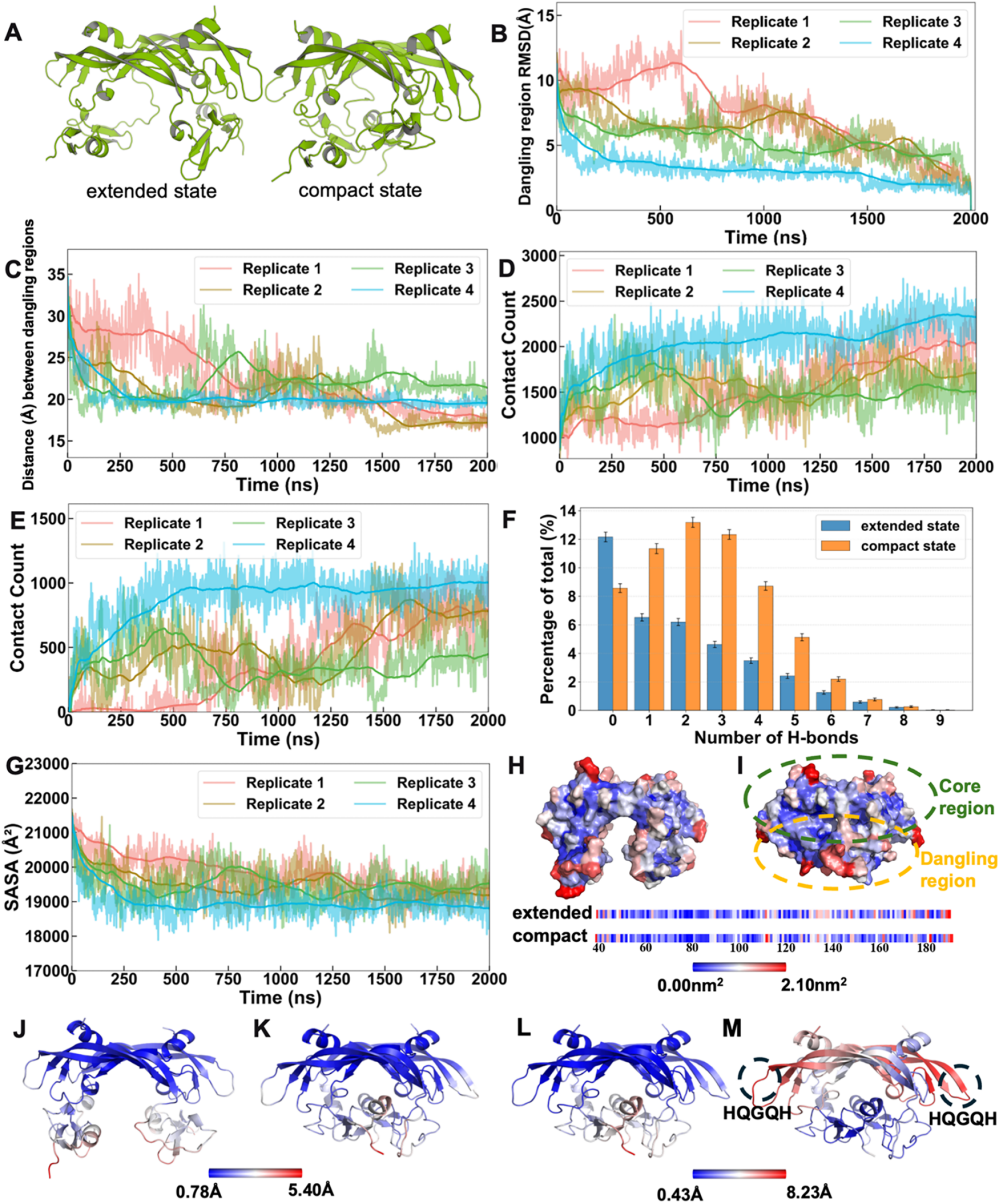
The conformational change of VEGF_165_. (A) Schematic representation of VEGF_165_ in the extended and compact states. (B) RMSD of the dangling regions, aligned to the final frame of VEGF_165_ from four 2000 ns MD simulation replicates. (C) Time evolution of the COM distance between the two dangling regions. (D) Number of contacts between the two dangling regions and the rest of the protein over time. (E) Number of contacts between the two dangling regions over time. (F) Percentage distribution of hydrogen bond numbers across four simulation replicates. Bars represent mean values from four independent simulations, and error bars represent bootstrap standard errors. The extended (blue) and compact (orange) states were defined based on structural analysis, with approximate time intervals of 0–750 ns and 750–2000 ns, respectively. (G) SASA of the entire protein over time. (H) SASA of VEGF_165_ in a representative extended state structure (0 ns). (I) SASA of VEGF_165_ in a representative compact state structure (2000 ns). (J) RMSF profile of the extended state (approximated by 0–750 ns). (K) RMSF profile of the compact state (approximated by 750–2000 ns). (L) RMSF mapped onto the VEGF_165_ structure, aligned using the core regions. (M) RMSF mapped onto the structure, aligned using the dangling regions. In B–E and G, raw data are shown as translucent lines and running averages are shown as bold lines.

To investigate the molecular interactions driving the observed conformational compaction, we computed the number of contacts formed over time. The number of contacts between the two dangling regions and the rest of the protein (Figure 1D) showed a gradual increasing trend. Similarly, the number of contacts between the two dangling regions themselves also increased over time (Figure 1E). Further analysis identified H-bonds as critical contributors to this conformational change. Specifically, in replicates 1 and 2, the number of H-bonds remained low (<2) during the initial stage, but subsequently increased to approximately four (Supporting Figure S2). A similar increasing trend was observed across all other replicates. The percentage distribution of H-bond numbers across four simulation replicates (Figure 1F) reveals a clear shift toward a higher number of H-bonds as the protein transitions from the extended to the compact state. To present the comparison between the two states, 750 ns is selected as a dividing time point when this transition finished based on visual inspection and the structural analyses. Specifically, we calculated the mean and standard deviation of key structural metrics pooled across all four replicates for the 0–750 ns and 750–2000 ns ensembles (Supporting Table S1). While all pairs of values fall within the corresponding standard deviations and therefore do not indicate statistically significant differences, we observe consistent directional changes across all metrics between the two phases (lower RMSD, lower COM distance, increased contacts, and reduced SASA), which may suggest the presence of a conformational transition after 750 ns. Concurrently, the SASA of the entire protein also showed a consistent decrease over the same time period (Figure 1G). This reduction in SASA suggests that the dangling regions and their surrounding residues become increasingly buried within the protein structure as these contacts form. Collectively, the concurrent increase in the number of contacts and H-bonds, alongside a reduction in solvent accessibility, indicates that the dangling regions undergo a dynamic conformational adjustment, progressively engaging in more interactions both internally and with the core protein.

To spatially visualize the extent of this compaction, SASA values of individual residues were projected onto the protein structure at 0 ns (representing the extended state) and 2000 ns (representing the compact state) (Figure 1H–I). This visualization clearly showed pronounced decreases in SASA for several residues within the dangling regions, confirming their burial upon compaction. Furthermore, to assess the changes in protein flexibility, we compared the average RMSF between the extended and compact states (Figure 1J–K). As in the previous analyses, 0–750 ns and 750–2000 ns are used as approximate time periods for the extended and compact states, respectively. The dangling region exhibited higher RMSF values in the extended state, indicating greater flexibility, which was visually represented by stronger fluctuations (red regions). Furthermore, average RMSF values were calculated from the 2000 ns trajectories, aligned either to the core region (Figure 1L) or the dangling region (Figure 1M), and subsequently projected onto the final protein structure. Notably, even when aligned to the dangling region, the end of the β sheet within the core region still demonstrated higher RMSF, suggesting persistent localized flexibility.

To identify key residues involved in the formation of interdangling region H-bonds, we analyzed the most frequent H-bonding residue pairs across trajectories. The top three pairs are summarized in Table 1. Notably, the four simulation replicates exhibit hydrogen bond patterns that are distinct yet share similarities. Specifically, the Arg136–Asp169 H-bond was predominantly formed in Replicate 3, while 3 out of 4 replicates showed almost no formation of this H-bond. Similarly, the Arg171–Asp157 H-bond was exclusively observed in Replicate 4, with 0% occupancy in the other three replicates. In contrast, the Lys173–Glu140 H-bond showed more consistent formation across three replicates, although it was absent in Replicate 4. Time-resolved distances between these specific residue pairs revealed their interaction dynamics (Figure 2A–D). A representative final snapshot illustrating this frequent H-bond pair (Lys173–Glu140) is shown in Figure 2E. The snapshot of other H-bond pairs is shown in Supporting Figure S3.

**Figure 2:**
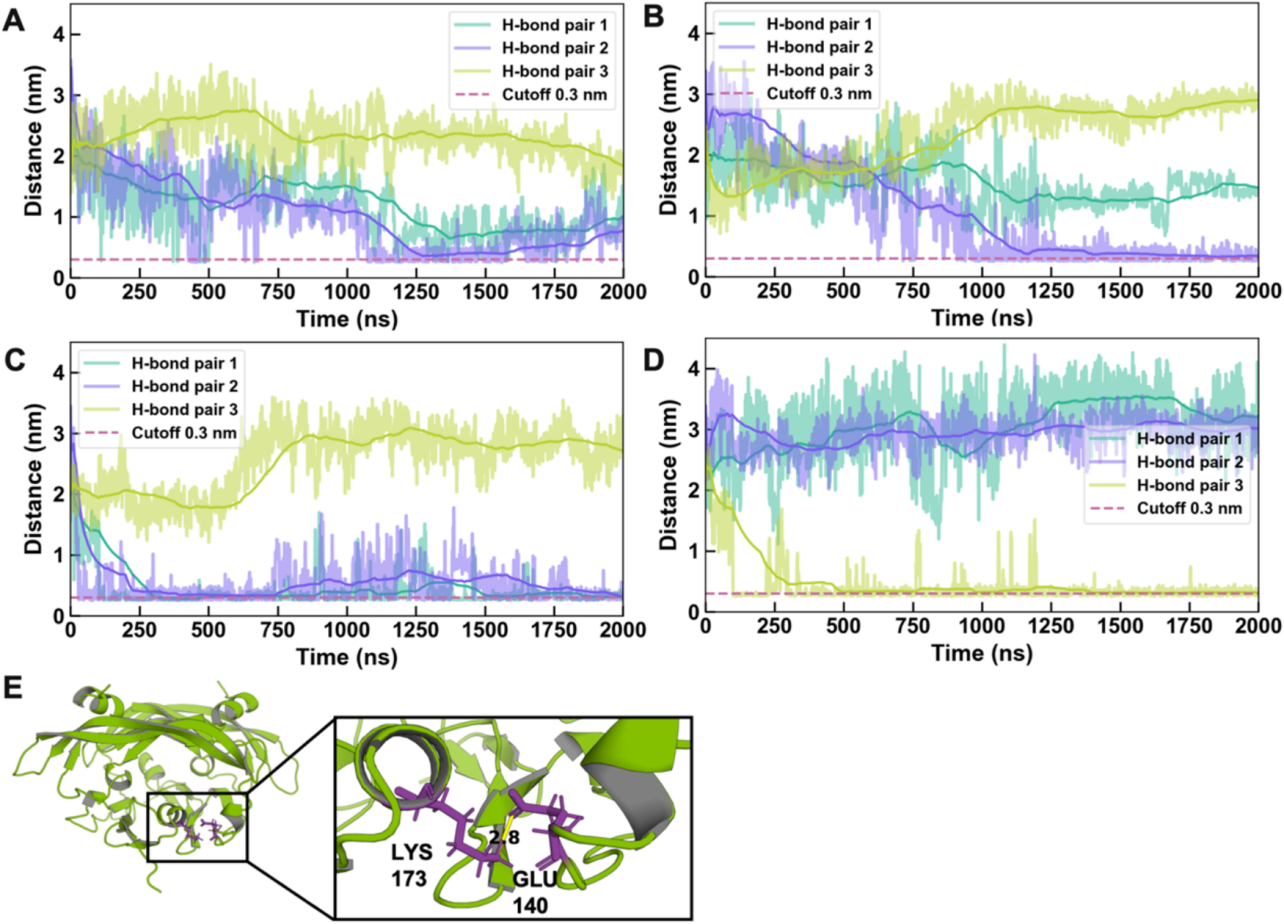
H-bonds of the VEGF_165_ during conformational change. (A–D) Time evolution of distances for the three most persistent H-bond pairs in four simulation replicates. (E) Snapshot of the final frame showing the H-bond pair (Lys173–Glu140) interaction between the two dangling regions. The distance between the nitrogen atom in the side chain of Lys173 and the oxygen atom in the side chain of Glu140 is 2.8 Å. In (A–D), raw data are shown as translucent lines and running averages are shown as bold lines.

**Table 1:** Three Most Frequently Formed Hydrogen Bond Pairs^a^

**donor**	**acceptor**	**average (%)**	**replicate 1 (%)**	**replicate 2 (%)**	**replicate 3 (%)**	**replicate 4 (%)**
Arg136	Asp169	19	1.9	0	74	0
Lys173	Glu140	18	21	19	33	0
Arg171	Asp157	14	0	0	0	58

a Percentages indicate the hydrogen bond occupancy over the 2000 ns simulation trajectories for four independent replicates.

### VEGF_165_ Binding to AuNPs

Having characterized the intrinsic conformational dynamics of VEGF_165_, we next investigated its potential interaction with nanoparticles. For this purpose, a fixed AuNP was introduced into the simulation system, as detailed in the Methods section and illustrated in Figure 3A. VEGF_165_ successfully attached to the AuNP surface in all 21 independent 200 ns MD simulations. Contact analysis across the frames where any protein residue was in contact with AuNP surface demonstrated that residues 112-116 (HQGQH), 136R, 149R, and 191R exhibited high contact frequencies with the AuNP surface (Figure 3B–C). Notably, the HQGQH represents a relatively extended motif with a consistently high contact ratio, whereas other identified interacting sites typically comprised single or paired residues. In conjunction with our earlier RMSF analysis, which indicated the flexibility in this region, these findings suggested that the HQGQH sequence constituted a key binding motif in binding to AuNPs. Additionally, a detailed structural visualization shows how residues 136R, 149R, and 191R interact with the AuNP surface, showing a cooperative binding model (Supporting Figure S5).

**Figure 3:**
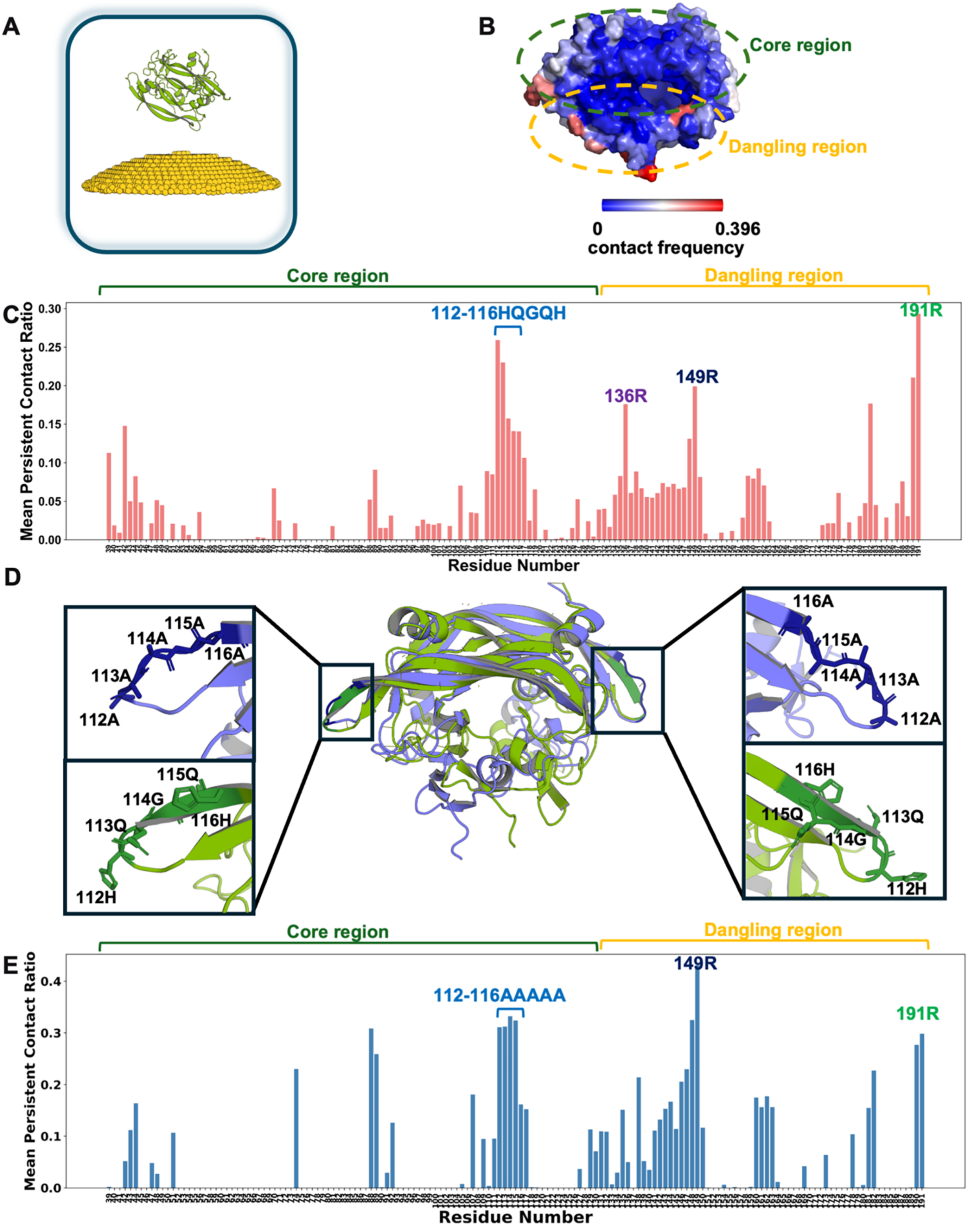
Model of the VEGF_165_-AuNP and the MD results. (A) Schematic representation of the simulation model: VEGF_165_ (splitpea green) with a spherical cap-shaped AuNP (golden). Water molecules, Na^+^, and Cl^–^ ions are omitted for clarity. (B) Average contact ratio between VEGF_165_ residues and the AuNP surface across the contact frames of the 21 independent simulations. (C) Contact ratio (cutoff 4.5 Å) mapped onto the final-state protein structure. (D) Structural alignment of VEGF_165_ (splitpea green) and its mutant (slate blue). (E) Average contact ratio between 5A mutant residues and the AuNP surface across the contact frames of the 3 independent simulations.

To investigate the binding behavior of the 5A mutant, we first introduced a 5A mutation (HQGQH to AAAAA) in the 112–116 region into our computational model of VEGF_165_ (Figure 3D). A comprehensive structural analysis of the 5A mutant compared with WT, including RMSD, RMSF and SASA, verifies that the mutation does not induce global structural perturbations (Supporting Figure S6). We then performed three independent unbiased MD simulations using the same protocol. Interestingly, the contact analysis of the 5A mutant revealed a contact pattern largely similar to that of the WT across the whole protein sequence (Figure 3E). This persistence of contact in unbiased simulations necessitated our subsequent PMF calculations to quantify the binding affinity differences.

To experimentally validate this computationally predicted binding motif between AuNPs and VEGF_165_, we introduced a 5A mutant (HQGQH to AAAAA) of VEGF_165_ and compared the binding characteristics of the WT with AuNPs against the 5A mutant. The 5A mutant was expressed in *E. coli* system, and its identity and molecular weight (19.9 kDa) were confirmed by Coomassie blue staining and Western blot (Supporting Figure S7). The AuNPs employed in this study were characterized by SEM, revealing a spherical morphology with an average diameter of approximately 20 nm (Supporting Figure S8).

ELISA was employed to quantify the concentration of unbound VEGF_165_ after incubation with AuNPs, thereby determining the binding rate, and the raw data were provided in Supporting Table S3. Our binding assays demonstrated that the binding rate between WT and AuNPs was 31 ± 4%, while the binding rate between the 5A mutant and AuNPs was only 5 ± 4% (Figure 4A). To further elucidate the importance of the identified motif, we also explored the ζ potential of the AuNPs in the presence and absence of VEGF_165_. AuNPs alone exhibited a ζ potential of −22 ± 1 mV. Upon incubation with the WT, the ζ potential of the AuNPs significantly increased to −14 ± 1 mV (Figure 4B), indicating substantial protein adsorption and charge neutralization. Crucially, when the AuNPs were incubated with the 5A mutant, the ζ potential only slightly increased to −20 ± 1 mV (Figure 4B), suggesting significantly weaker or negligible protein binding. Our ELISA assays and ζ potential shifts both support the involvement of the identified motif in mediating the interaction between VEGF_165_ and AuNPs.

**Figure 4:**
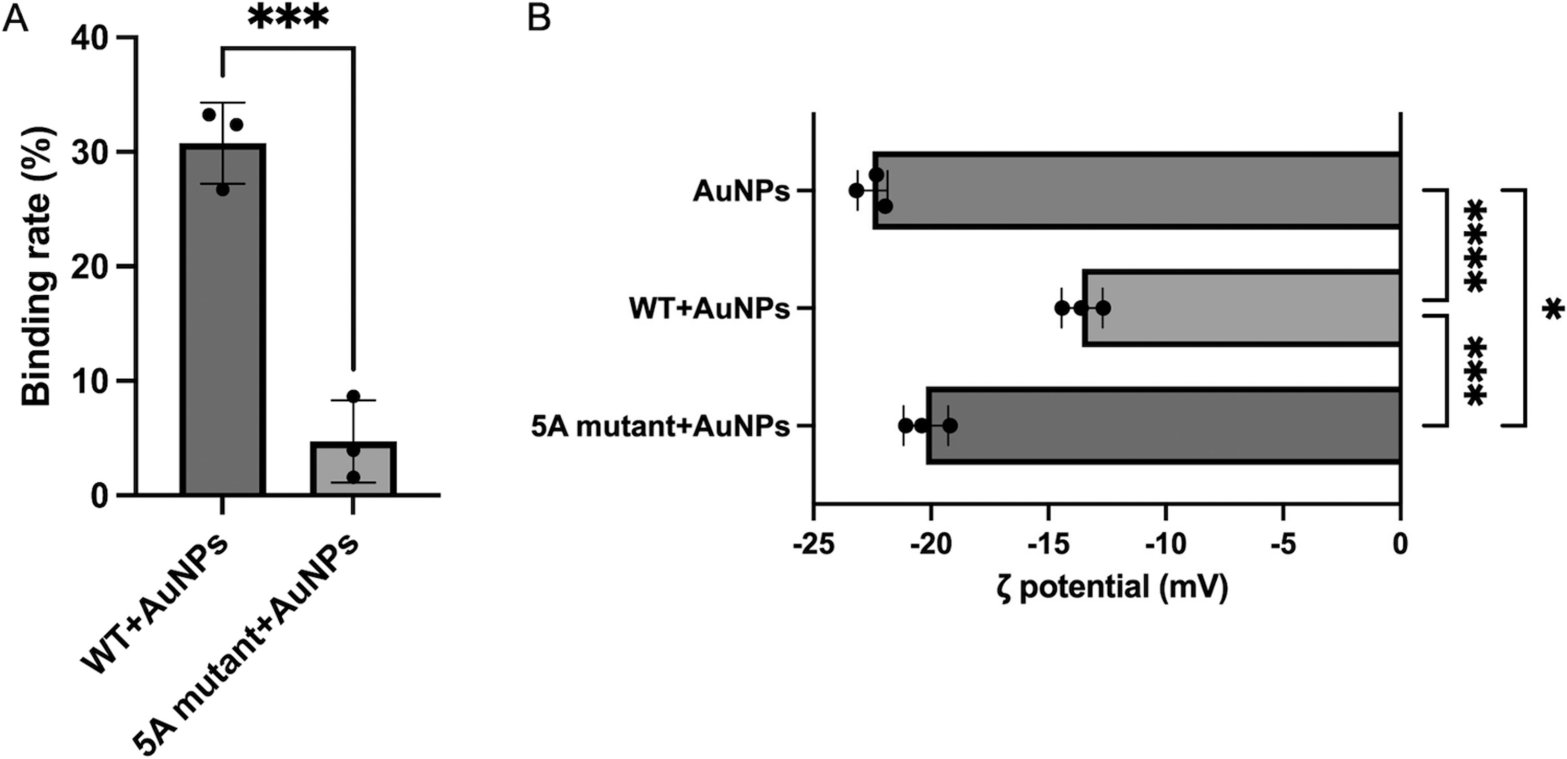
Comparison of the binding between WT and AuNPs versus 5A mutant and AuNPs. (A) Binding rate of WT/AuNPs and 5A mutant/AuNPs calculated from ELISA measurements. Statistical significance was determined by an unpaired *t* test. (B) ζ potential of WT/AuNPs and 5A mutant/AuNPs. Statistical significance was determined by one-way ANOVA (Šídák’s multiple comparisons test). Data presented as means ± SD *p < 0.05, **p < 0.01, ***p < 0.001, ****p < 0.0001.

To quantitatively validate the role of the HQGQH motif, we also introduced the 5A mutant into our computational model of VEGF_165_ (Figure 3D). We conducted SMD simulations for both the WT and 5A mutant to probe their unbinding pathways and forces (Figure 5A). Initial SMD simulations from random starting conformations produced varied pulling force profiles, which were expected due to the multiple potential binding sites on the AuNP surface (Supporting Figure S9). To ensure a direct comparison of the HQGQH motif’s contribution, we selected a representative trajectory where this specific motif was initially bound to the AuNP surface. The final conformation from this trajectory was then used as the starting structure for focused SMD simulations on both the WT and 5A mutant. On average, the WT system detached at a farther distance (∼6.0 nm) than the 5A mutant (∼5.3 nm) (Figure 5B). Detailed pulling force profiles are provided in Supporting Figure S10.

**Figure 5:**
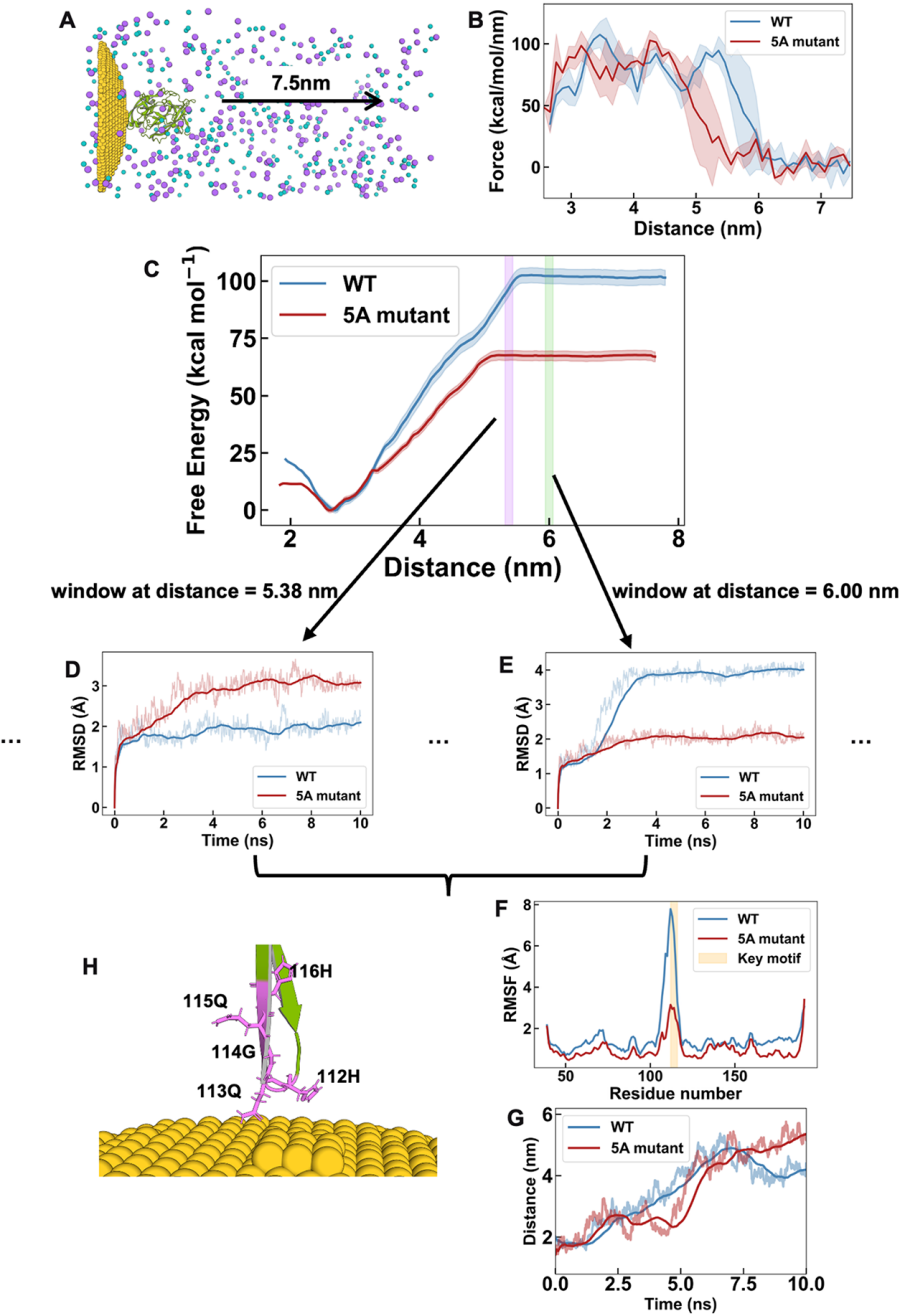
SMD and US. (A) Schematic illustration of the SMD experimental setup: VEGF_165_ was pulled approximately 4.6 nm away from the AuNP. Blue and purple spheres denote Na^+^ and Cl^–^ ions, respectively. Water molecules are omitted for clarity. (B) Pulling force profiles comparing WT and 5A mutant when only the HQGQH/AAAAA motif was initially involved in binding. Data is represented as mean (solid line) and standard deviation (shaded band) from three independent replicates. (C) PMF from US for WT and 5A mutant. (D) RMSD of the WT and 5A mutant during the US window at a protein–AuNP distance of 5.38 nm. (E) RMSD of the WT and 5A mutant during the US window at a protein–AuNP distance of 6.00 nm. (F) RMSF of chain B in WT and 5A mutant, taken from the respective US windows where each protein detached from the AuNP. (G) Time evolution of the distance between a representative Au atom and the Cα COM of the key motif residue 112-116 (HQGQH). (H) Snapshot from the SMD trajectory showing the final residues of WT detaching from the AuNP. In D, E and G, raw data are shown as translucent lines and running averages are shown as bold lines.

To obtain a more quantitative and thermodynamically robust ranking of binding affinity, US was employed to calculate the potential of mean force (PMF). The binding free energy for the WT was determined to be 102.66 kcal/mol, whereas the 5A mutant showed a significantly reduced value of 67.78 kcal/mol (Figure 5C). It should be noted that although both values represent favorable binding energies in absolute terms, the lower ranking for the 5A mutant indicates the critical role of the HQGQH motif in mediating the interaction between VEGF_165_ and AuNPs. The calculated binding free energies for both WT and 5A mutant stabilized after US window simulation at approximately 5.5 and 5.1 nm, respectively (Figure 5C). To confirm the reliability of these profiles, bootstrap analysis was performed to assess statistical uncertainty, which is represented by the shaded error bands in Figure 5C. The adjacent US windows showed sufficient overlap in their umbrella histograms (Supporting Figure S11A). Additionally, the PMF profiles derived from the sequential time blocks (i.e., 0–2 ns, 0–4 ns, 0–6 ns, 0–8 ns, and 0–10 ns) eventually overlay, ensuring reliable PMF reconstruction via WHAM (Supporting Figure S11B). It is worth noting that while the PMF results indicate a clear reduction in binding free energy for the 5A mutant, the average peak force from SMD shows only a minor difference between the two systems (Figure 5B). This discrepancy may stem from the inherent differences between these two methods. PMF is derived from equilibrium sampling and generally provides a more robust measure of thermodynamic stability, whereas SMD probes nonequilibrium constant forces that are influenced by factors like pulling speed and pathway-specific energy barriers. Nevertheless, the consistent trend across both methods supports weakened binding upon mutation, with the PMF offering a definitive thermodynamic validation.

Further structural insights into the unbinding process were obtained from the analysis of US simulation trajectories. In a US window with a protein–AuNP separation distance of 5.38 nm, the 5A mutant gradually lost direct contacts with the AuNP surface. Importantly, the protein did not undergo large structural deformation during this detachment process; its global fold remained stable. The larger RMSD values observed over time (Figure 5D) primarily reflect the increased orientational freedom and conformational flexibility of the protein once it was no longer tethered to the nanoparticle surface. In contrast, the WT remained stably bound at this distance (Figure 5D). Conversely, in a US window at a constrained COM separation distance of 6.00 nm, the WT protein, while initially bound, progressively dissociated, leading to larger RMSD values. At this same distance, the 5A mutant had already detached and exhibited stable, low RMSD values throughout the simulation (Figure 5E). A comparison of the RMSF for chain B of the WT and 5A mutant, taken from their respective US windows corresponding to unbinding, highlights key differences (Figure 5F). The residues within the HQGQH motif (112-116) displayed higher RMSF values compared to other regions in both systems, indicating their high flexibility. Notably, the WT exhibited greater fluctuations than the 5A mutant across the whole chain B and particularly in the key motif itself. These results indicated more pronounced conformational changes and ongoing dissociation in this region for the WT at this stage. The time evolution of the distance between a representative Au atom and the Cα COM of the key motif residue 112-116 (HQGQH) confirmed these findings (Figure 5G). The WT trajectory showed a smoother increase in distance from approximately 2 to 6 nm, indicating a more continuous detachment process. In contrast, the steeper and shorter increase observed for the 5A mutant suggested a more abrupt detachment from the AuNP. To further resolve the molecular details of this process, Figure 5H presents a snapshot from the SMD trajectory highlighting the final residues of WT that disengaged from the AuNP. The last residue to dissociate was Q113, followed by H112.

### The HQGQH Motif Mediates VEGF_165_ Binding to AuNPs and Impairs VEGF_165_-Induced Endothelial Cell Migration

To validate that the HQGQH motif is critical for this interaction experimentally, we compared the effects of AuNPs on endothelial cell migration and angiogenesis induced by the WT and the 5A mutant. First, we assessed the cytotoxicity of AuNPs alone. Treatment with 5 μM AuNPs resulted in low cytotoxicity, with mean cell viability decreasing to 83 ± 1% (Supporting Figure S12). We then evaluated VEGF_165_-induced endothelial cell migration in the presence of AuNPs. As shown in Figure 6A, relative to the negative control (NC) group, WT increased the number of migrated endothelial cells from 384 ± 55 to 583 ± 32, a stronger effect than that observed with the 5A mutant (from 384 ± 55 to 505 ± 55). The addition of 5 μM AuNPs reduced WT-induced cell migration by 52% (from 583 ± 32 to 282 ± 30) and 5A mutant-induced migration by 38% (from 505 ± 55 to 312 ± 15), demonstrating that AuNPs exert a stronger inhibitory effect on WT than on the 5A mutant. These results confirm that the HQGQH motif plays a key role in VEGF_165_/AuNPs binding and the subsequent inhibition of VEGF_165_-induced cellular responses.

**Figure 6:**
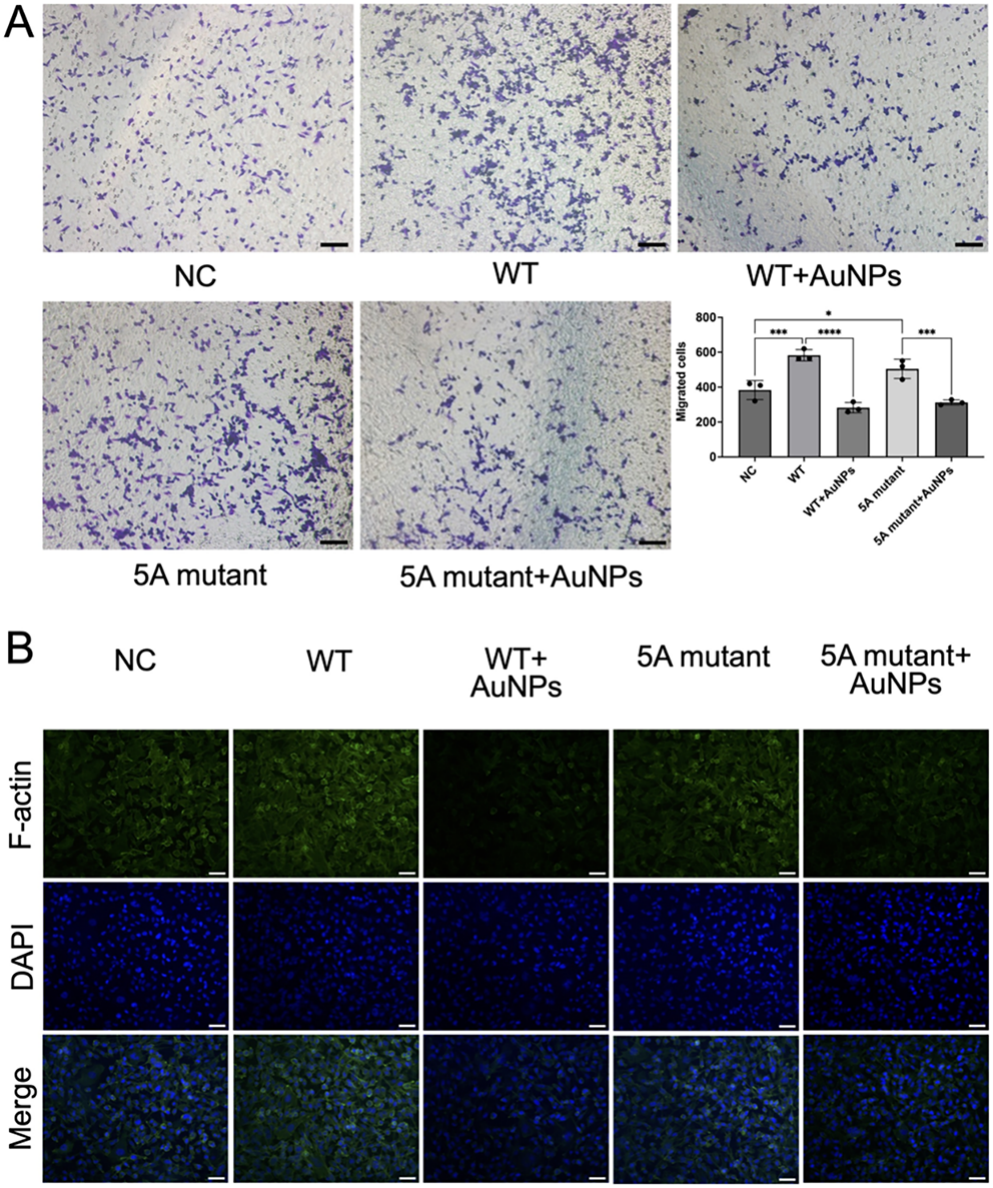
The critical involvement of the HQGQH motif in AuNPs’ inhibition on VEGF_165_-induced cell migration. (A) Transwell assay of hCMEC/D3 cells for the comparison of the inhibition effects of AuNPs on WT-induced cell migration and such inhibition on 5A mutant-induced cell migration. Representative micrographs showed cells that migrated through the transwell membrane, visualized by purple staining. The bar graph quantifies the number of migrated cells per field. Data presented as means ± SD. Statistical significance was determined by one-way ANOVA (Šídák’s multiple comparisons test). Scale bar = 100 μm. (B) F-actin immunofluorescence staining of hCMEC/D3 cells after the indicated treatments. Scale bar = 50 μm.

Consistent with the migration data, F-actin staining revealed parallel changes in cytoskeletal dynamics (Figure 6B). Cells in the NC group exhibited a basal F-actin expression level, and WT treatment led to a tendency of increased expression, reflecting active cytoskeletal reorganization. The cotreatment of WT and AuNPs decreased such a tendency. The 5A mutant group also had a tendency for enhanced F-actin expression compared to NC, but to a lesser degree than that in the WT group. Additionally, the cotreatment of 5A mutant and AuNPs decreased this tendency, but the degree of this decrease was less, reflecting HQGQH-dependent actin remodeling to AuNPs inhibition effects. These results indicated that the inhibitory effect of AuNPs on actin remodeling was dependent on the HQGQH motif.

### The Significance of the HQGQH Motif in the Inhibition Effects of AuNPs on Cell Angiogenesis

The angiogenesis assay (Figure 7A) further supported the importance of the HQGQH sequence and revealed its relevance to the inhibitory effect of AuNPs on cell angiogenesis. At 8 h, cells in the NC group only formed sparse and rudimentary networks with the mean total tube length of 6954 ± 546 μm. Instead, cells in the WT group developed extensive, well-branched capillary-like structures with a mean total tube length of 13,804 ± 2488 μm, indicating the critical role of VEGF_165_ in promoting cell angiogenesis. For the WT and AuNPs cotreatment group, the pro-angiogenic ability of WT dropped by approximately 59%, where the mean total tube length was only 5706 ± 295 μm. 5A mutant also promoted angiogenesis, while the networks in the 5A mutant group were consistently less robust than those in the WT group, with shorter tube length of 10,854 ± 1206 μm with fewer branches, indicating that the HQGQH mutation attenuated the angiogenic potency of VEGF_165_. Co-treatment of 5A mutant and AuNPs led to less extensive networks, where the mean tube length decreased by around 35%. It is noteworthy that AuNPs had stronger inhibition effects on WT-induced cell angiogenesis than that on 5A mutant-induced cell angiogenesis, probably because of the binding between AuNPs and HQGQH motif in WT. These observations were corroborated by CD31 immunostaining (Figure 7B). In the WT group, cells showed a tendency of intense CD31 expression and clear cell–cell junctions outlining vessel-like structures, while the cotreatment of AuNPs greatly decreased such tendency. The 5A mutant induced a tendency of a moderate increase in CD31-positive network formation, and the cotreatment of AuNPs greatly decreased such tendency similarly. These findings demonstrated that the HQGQH motif had pro-angiogenic effects on tube formation, and AuNPs suppressed such a process, indicating the robust inhibition of AuNPs on WT-induced cell angiogenesis.

**Figure 7:**
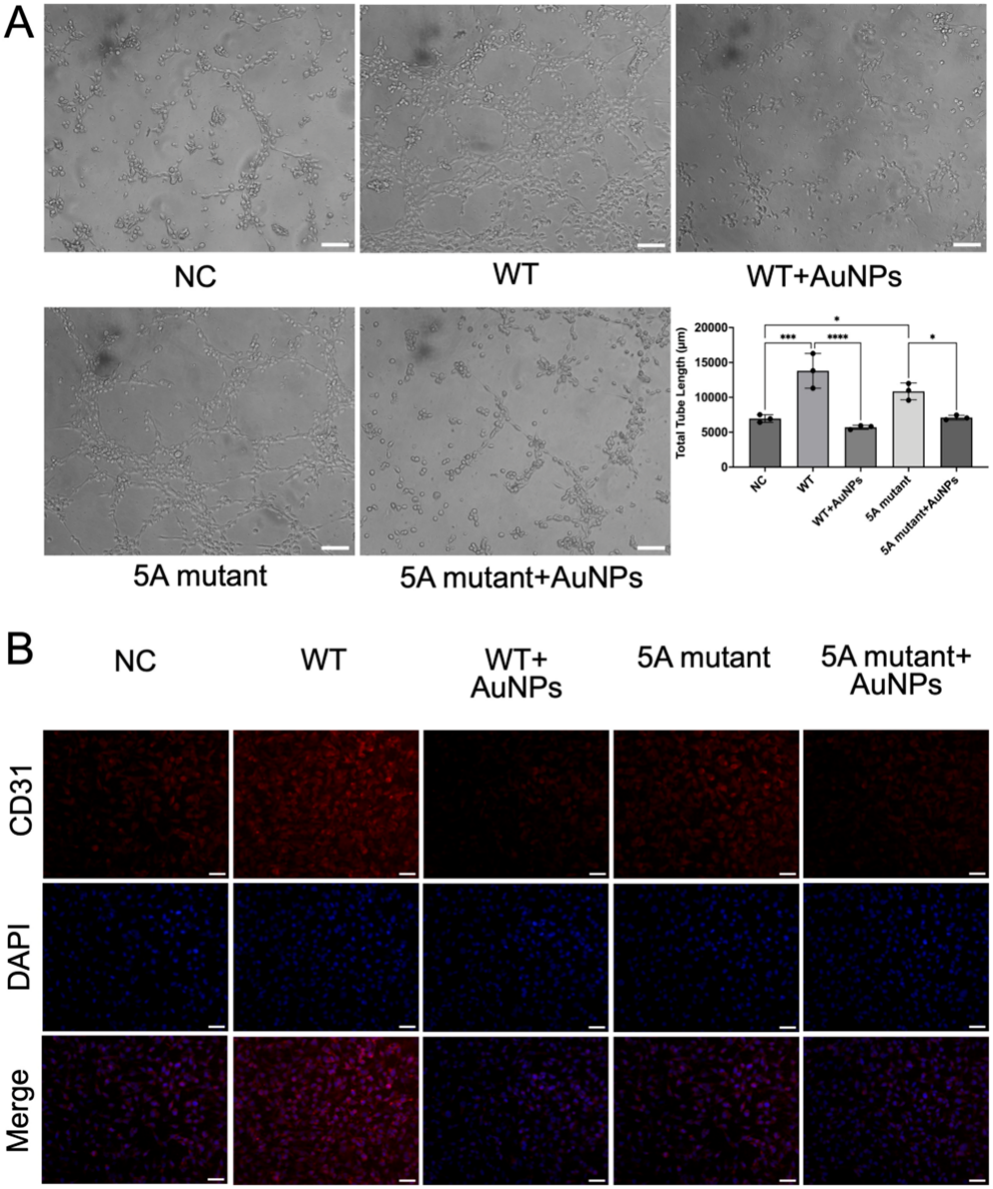
The critical involvement of the HQGQH motif in AuNPs’ inhibition on VEGF_165_-induced cell angiogenesis. (A) Angiogenesis assay of hCMEC/D3 cells for the comparison of the inhibition effects of AuNPs on WT-induced cell angiogenesis and such inhibition on the 5A mutant-induced cell angiogenesis. Data presented as means ± SD. Statistical significance was determined by one-way ANOVA (Šídák’s multiple comparisons test). Scale bar = 100 μm. (B) CD31 immunofluorescence staining (red) of hCMEC/D3 cells after 8 h of tube formation. Scale bar = 50 μm.

## Discussion

This study identified the HQGQH motif as the critical motif responsible for VEGF_165_/AuNPs binding through MD simulations. The binding efficiency at this site was experimentally validated, along with the subsequent decline in the functional role of the HQGQH motif upon VEGF_165_/AuNPs complex formation. MD simulations revealed that VEGF_165_ underwent conformational transitions between extended and compact states, driven by intramolecular interactions involving its flexible terminal regions. Analysis of molecular contacts identified the HQGQH motif (residues 112-116) as the predominant site responsible for AuNPs interaction. Substitution of this motif from HQGQH to AAAAA significantly weakened the interaction, as evidenced by PMF calculations showing reduced binding free energy for the 5A mutant. ELISA and ζ potential measurements confirmed that WT exhibits a stronger binding affinity to AuNPs compared to the 5A mutant. Migration and angiogenesis assays further confirmed that the binding between the HQGQH motif and AuNPs inhibited the VEGF_165_-induced cell migration and angiogenesis. It is important to note that the PMF calculations rank rather than quantitatively measure absolute binding affinities. As both the WT and 5A mutant show high computed binding free energies, the simulations do not predict the mutant to attach weakly as seen from experiments. However, the substantial reduction in binding energy upon mutation correctly ranks the relative affinities, demonstrating that our computational approach is reliable for comparing relative binding strengths despite the overestimation of absolute thermodynamic magnitudes.

Methodologically, our long-time scale MD simulations (2000 ns) addressed the limitations of prior short-duration studies, enabling *in silico* capture of the complete conformational transition of VEGF_165_ from an extended to a compact state. This finding aligns with anisotropic network model predictions of reduced inter-HBD distances.^29^ Crucially, this spontaneous change occurred in the absence of heparin, highlighting its intrinsic structural dynamics. While this compaction parallels observations that heparin binding increases solvent exposure of nonpolar regions,^34^ the mechanistic relationship remains unresolved, as it is unclear whether heparin potentiates or opposes HBD convergence and how such modulation alters functional outcome.

Functionally, this conformational shift carries significant biological implications. In the extended state, the C-terminal HBDs exhibit high flexibility with elevated SASA, facilitating initial interactions with heparan sulfate proteoglycans in the extracellular matrix (ECM). This configuration likely promotes VEGF_165_ anchoring, prolonging local bioavailability and establishing chemotactic gradients to direct endothelial cell migration.^9^ Conversely, in the compact state, intermonomer H-bonds (e.g., Arg136–Asp169 and Lys173–Glu140) stabilize HBD proximity, reducing SASA and potentially modulating VEGFR-2 binding affinity. This dynamic adaptability allows VEGF_165_ to fine-tune angiogenic responses: the extended state favors ECM retention and gradient formation, whereas the compact state may optimize receptor activation efficiency during vessel maturation.

We found the HQGQH motif functions as a critical structural determinant for VEGF_165_/AuNPs binding, and mutation of this motif further verified the essential role of this motif in mediating VEGF_165_/AuNPs interactions. Similarly, a previous study found the single mutation of G88C altered ζ potential values in VEGF/AuNPs binding.^24^ Interestingly, G88 corresponds to the third residue within the HQGQH motif identified in our study. We also found that the binding between AuNPs and VEGF_165_ through the HQGQH motif significantly inhibited VEGF_165_-induced cell migration and angiogenesis. The HQGQH motif remains in the RBD to promote VEGF_165_/VEGFR-2 binding and stimulate downstream signals for angiogenesis. We inferred that the binding between VEGF_165_ and AuNPs could reduce the free VEGF_165_ in the microenvironment. Consequently, less VEGF_165_ was left to promote the migration and angiogenesis of endothelial cells, leading to a decrease in VEGF_165_-induced cell migration and angiogenesis after AuNPs treatment. In detail, Ile83, Lys84, and Pro85 of VEGF are part of the largest hot spot in VEGF/VEGFR-2 binding, and HQGQH is spatially close to these amino acids.^35^ Similarly, another study found His86 of VEGF-A can interact with the domains 2 and 3 of VEGFR-2, where His86 is the first His in HQGQH motif. Thus, VEGF_165_/AuNPs binding on the HQGQH motif may spatially disrupt VEGF_165_/VEGFR-2 binding, thus inhibiting pro-anagenetic signals. In contrast, according to a heparin-Sepharose precipitation experiment, AuNPs bind to the HBD of VEGF_165_.^18^

In our work, HQGQH in RBD is a main and critical motif in VEGF_165_/AuNPs interactions, while HBD may also contribute to binding. Interestingly, VEGF_121_ also has the HQGQH motif, while AuNPs could not bind to VEGF_121_ and failed to inhibit cell proliferation mediated by VEGF_121_.^36^ We think AuNPs could directly bind to the HQGQH motif in RBD, and the HBD may have a cooperative function in this process, as HQGQH is spatially close to the HBD. It is noteworthy that, relative to the NC group, the 5A mutant still promoted cell migration (Figure 6A) and angiogenesis (Figure 7A), but to a lesser degree than the WT. These differences can be attributed to the mechanism of VEGF_165_/VEGFR-2 interactions. The binding of VEGF_165_ to its primary receptor VEGFR-2 is an interfacial process involving multiple binding motifs. Even after mutation of one binding region (HQGQH to AAAAA), other motifs likely remain functional and capable of interacting with VEGFR-2 to promote cell migration and angiogenesis. While HQGQH is an important motif in VEGF_165_/AuNPs interactions, it may not be the most critical determinant of VEGF_165_/VEGFR-2 binding. Besides, a more modest inhibition of VEGF_165_-induced cell migration was observed for the 5A mutant/AuNPs combination (38% decrease) compared to WT/AuNPs (52% decrease) (Figure 6A). This differential inhibition likely arises from AuNP-mediated disruption of VEGF_165_/VEGFR-2 interactions. Simulation results suggest that HQGQH serves as the primary binding motif for AuNPs on VEGF_165_. For the WT, AuNPs bind preferentially to this site, thereby interacting with a larger number of WT molecules. In contrast, mutation of the HQGQH motif weakens AuNP binding to the 5A mutant, leaving more unbound 5A mutant available for VEGFR-2 interaction.

This study establishes a multiscale framework demonstrating synergistic integration of computational and experimental methodologies, but it still has some limitations. RMSF profiling and contact ratio calculations initially identify the 112-116 (HQGQH) motif as a primary AuNP-binding site. Subsequently, this is validated by ELISA binding assays and ζ potential shifts. However, quantitative SMD simulations revealed greater mechanistic complexity. When forcibly dissociating VEGF_165_ from AuNP surfaces, we observed that, in addition to HQGQH, multiple alternative residues mediated persistent interactions, with some exhibiting unbinding forces comparable to the motif. In specific trajectories, residues from the dangling region (e.g., Arg147, Lys150, and Arg182) remained bound to the AuNP surface, generating even larger pulling forces (Supporting Figure S9). These findings support a cooperative binding model in which HQGQH functions as the primary recognition site, while additional spatially distributed residues provide complementary contributions that stabilize the interaction.

Future studies should quantitatively resolve the hierarchical contributions of these residues from the dangling region to better determine their significance in VEGF_165_/AuNPs binding. Additionally, though we have found the binding motif and its significance in tube formation by its interactions with AuNPs, many other proangiogenic factors could promote tumor angiogenesis. It is promising to modify AuNPs for targeting proangiogenic factors to inhibit tumor angiogenesis in preclinical studies in the future.

## Conclusion

In conclusion, our findings demonstrated that the HQGQH motif plays an essential role in VEGF_165_/AuNPs binding. Mutation of this motif significantly diminishes VEGF_165_ binding affinity to AuNPs and attenuates VEGF_165_-mediated migration and angiogenesis. This study elucidates the underlying mechanism of VEGF_165_/AuNPs binding and provides new insights for the development of potential antiangiogenic therapies.

## Methods

### VEGF_165_ Mutant

The wild type of recombination VEGF_165_ protein (P15692-4) was purchased from Sino Biological (China). The mutated recombination VEGF_165_ protein was generated by substituting the HQGQH motif into AAAAA, with a His tag at the N-terminal of the protein, by *E. coli*. The mutated recombination vector was transformed into *E. coli*. strain BL21, and then 0.2 mM of isopropyl-ß-d-thiogalactopyranoside (Solarbio, China) was used to induce the expression of the recombinant fusion protein at 16 °C for 18 h. The 5A mutant was further purified by nickel-nitrilotriacetic acid column (Smart-Lifesciences, China), washed with binding buffer (20 mM Tris, 0.5 M NaCl, and 0 mM imidazole), and subsequently eluted with elution buffer (20 mM Tris, 0.5 M NaCl, and 500 mM imidazole), followed by dialyzing in Tris saline buffer (20 mM Tris, 0.5 M NaCl, and pH of 8.0).

### Coomassie Blue Staining & Western Blotting

Coomassie blue staining and Western blotting were used to identify the 5A mutant. The electrophoresis was performed on a 4–15% acrylamide gel (Doyobio, China). Coomassie blue staining was performed using Coomassie Blue fast staining solution (Beyotime, China). For Western blotting, the gel was transferred onto a polyvinylidene difluoride membrane, which was subsequently blocked by 5% bovine serum albumin (BSA). The primary antibody was 1:10,000 dilution of 6*His, His-Tag monoclonal antibody (Proteintech, China) and the second antibody was 1:10,000 dilution of IRDye 800CW Donkey anti-Mouse IgG antibody (LI-COR, USA). The film was scanned with an imaging densitometer (Bio-Rad, USA).

### Scanning Electron Microscopy (SEM)

The morphology of AuNPs was determined by SEM. AuNPs solution (Zhongkekeyou, China) was dropped onto the copper net and dried at room temperature overnight. Imaging was performed by an HT7700 transmission electron microscope (Hitachi, Japan) with an accelerating voltage of 100 kV and magnification of 60 kX.

### ELISA

ELISA was used to measure the concentrations of unbound VEGF_165_ after the incubation of AuNPs and VEGF_165_, and the binding rate of VEGF_165_ and AuNPs was calculated by using the following formulas.

1 TotalVEGF165=boundVEGF165+unboundVEGF165

2 $$\mathrm{Binding}\;\mathrm{rate}=\frac{\mathrm{Total}\;\mathrm{VEGF}165-\mathrm{unbound}\;\mathrm{VEGF}165}{\mathrm{Total}\;\mathrm{VEGF}165}$$

For sample preparation, 600 μL of 80 ng/mL VEGF_165_ in PBS was mixed with 600 μL of 10 μM AuNPs in PBS or 600 μL of PBS. Thus, the working concentration of VEGF_165_ was 40 ng/mL, and that for AuNPs was 5 μM. The mixture was incubated at 25 revolutions per minute (rpm) at 4 °C before centrifuging at 1000*g* for 20 min. The supernatant was taken for ELISA detection by Human VEGF_165_ ELISA Kit (Elabscience, China). Briefly, 100 μL of the samples was sequentially incubated with antibody, HRP, substrate solution, and stop solution. Then the samples were measured under 450 nm by the SpectraMax 190 plate reader (Molecular Devices, China).

### ζ-potential Measurement

For sample preparation, 600 μL of 80 ng/mL VEGF_165_ in PBS was mixed with 600 μL of 10 μM AuNPs in PBS or 600 μL of PBS. The mixture was incubated at 25 rpm at 4 °C before surface charge detection. The ζ potential of the charged AuNPs was detected by the Zetasizer Nano ZS (Malvern Instruments, UK).

### Cell Viability Assay

Human Cortical Microvessels Endothelial Cells/D3 (hCMEC/D3) cells (Fuheng, China) were cultured in complete endothelial cell medium (ScienCell, USA) until reaching 80% confluency prior to the cell viability assay. For each well, 1 × 10^4^ hCMEC/D3 cells in 100 μL complete medium were cultured overnight. Cells were then exposed to complete medium with or without 5 μM AuNPs for 24 h. Subsequently, 10 μL of CCK-8 reagent (Biosharp, China) was added to each well and incubated for 1 h. Absorbance at 450 nm was recorded with a microplate reader, and background blank values were subtracted. Cell viability (%) was expressed relative to the negative control (NC) using Viability = (OD_sample_–OD_blank_)/(OD_NC_–OD_blank_) × 100%.

### Migration Assay

The migration of hCMEC/D3 cells was assessed in 24-well Boyden chambers fitted with 8 μm-pore inserts. For each well, 5 × 10^4^ hCMEC/D3 cells in 200 μL serum-free medium (0% FBS) were seeded on the upper chamber for 4 h for cell attachment. Then, 800 μL treatment medium was added into the lower chamber (Supporting Table S2) for 24 h. After fixation with 4% paraformaldehyde, staining with 0.1% Crystal violet dye, and washing with Milli-Q water, migrated cells were imaged through the Inverted Fluorescence Microscope from Nikon (Japan).

### Immunofluorescence of F-actin and CD31

Immunofluorescence of F-actin was assessed in a 24-well plate. A sterile glass coverslip was placed into each well, followed by seeding of 1.2 × 10^5^ hCMEC/D3 cells onto the coverslip in the well. After culturing overnight, cells were treated with 500 μL treatment medium for 24 h (Supporting Table S2). After fixing with 4% paraformaldehyde, permeabilizing with 0.1 % Triton X-100, and blocking with goat serum (Biosharp, China), samples were labeled with the CoraLite Plus 488-conjugated Phalloidin antibody (Proteintech, China) and DAPI (Beyotime, China). For immunofluorescence of CD31, cells were seeded similarly, and then fixed with 4% paraformaldehyde and blocked with goat serum. Afterward, samples were labeled with mouse antihuman CD31 antibody (1:200) (Proteintech, China), followed by ABflo 594-conjugated goat antimouse IgG (1:400) (Abclonal, China), and with DAPI. Images were captured using the Inverted Fluorescence Microscope (Nikon, Japan).

### Angiogenesis Assay

First, hCMEC/D3 cells were seeded in a 6-well plate to reach 80% confluency, and then cultured with treatment medium (Supporting Table S2) for 24 h, followed by cell starvation (0% FBS) for 12 h. Afterward, cells in each treatment group were digested, respectively. 12 μL of Matrigel (Corning, USA) was solidified in each well in a 24-well plate, and cells were seeded and cultured on the Matrigel in the wells, with 3 × 10^4^ cells in each well. Images were taken after culturing for 8 h by a microscope (Nikon, Japan), and total tube length was quantified using ImageJ.

### Standard Molecular Dynamics Simulation Setup

Atomic structure of the WT was obtained using AlphaFold2.^37^ VEGF_165_ is a homodimer composed of two identical chains. For each chain, residues 39-130 are defined as the core region, whereas the C-terminal tail (residues 131-191) is defined as the dangling region. The 5A mutant was modeled by making a point mutation in the WT protein structure using PyMOL.^38^ The 20 nm-diameter AuNP is modeled as a crystalline surface using CHARMM-GUI,^39^ due to its larger exposed surface and larger size compared to the protein.^40^ Meanwhile, considering the concentration of VEGF_165_ is higher than that of the larger AuNPs in the experimental setup, each system was configured to contain one VEGF_165_ molecule and one AuNP. All the gold atoms were fixed during all simulations. For the system containing VEGF_165_ alone, four independent 2000 ns molecular dynamics trajectories were collected for subsequent analysis. For systems involving VEGF_165_ in complex with AuNP, 21 independent 200 ns simulations were performed for WT, and 3 independent 200 ns simulations were performed for the 5A mutant. Specifically, the initial orientation of VEGF_165_ across these simulations was adjusted in PyMOL while keeping the AuNP existing and fixed for simplicity, with the initial distance between the two components maintained at >15 Å. Three representative starting structures of WT VEGF165 and AuNP is presented in Supporting Figure S4. One VEGF_165_ was solvated into a water box of 9.4 nm × 9.4 nm × 9.4 nm containing 24,869 water molecules. One VEGF_165_ and one AuNP were solvated into a water box of 14.4 nm × 14.4 nm × 14.4 nm containing 92,701 water molecules. In both cases, Na^+^ and Cl^–^ ions were added to the solvent to neutralize systems with 0.10 mol/L NaCl. Periodic boundary conditions were applied in all spatial directions.

### Steered Molecular Dynamics Simulation Setup

All AuNP atoms were fixed as in previous MD simulations, while an external force was applied to the core region of the VEGF_165_. After equilibration under physiological conditions, the atoms of the target residues were pulled away from the nearest AuNP atom at 0.0008 nm/ps with a spring constant of 1000 kJ·mol^–1^·nm^–2^. Three independent MD replicas were performed for each system configuration: (1) WT/AuNP with randomized initial conformations derived from previous standard MD simulations; (2) WT/AuNP with a key motif-bound initial conformation, also selected from a final-state frame of the previous simulations; (3) 5A mutant/AuNP with a mutated motif-bound initial conformation.

### Molecular Dynamics Simulation

All the MD simulations were carried out using the GROMACS-2023.2 software package^41^ with CHARMM36m Force Field.^42^ The TIP3P water model was adopted for solvent molecules.^43^ Long-range electrostatic interactions were conducted with the particle mesh Ewald method.^44^ The vdW interactions were calculated with a smooth cutoff distance of 1.2 nm. Each solvated system was first minimized using the steepest descent minimization method, followed by a 500 ps NVT annealing and a 1 ns NPT relaxation at 310 K and 1 bar. During production runs, the simulation temperature and pressure were fixed at 310 K and 1 bar with the v–rescale thermostat^45^ and Parrinello–Rahman coupling scheme,^145^ respectively. A time step of 2.0 fs was used with all bonds involving hydrogen atoms constrained using the LINCS algorithm, and coordinates were collected every 2 ps.

### Umbrella Sampling

The distance between the center of mass (COM) of the core region of the VEGF_165_ and the nearest Au atom was chosen as the reaction coordinate. A series of windows, 78 for WT and 65 for the 5A mutant, is evenly located along the reaction coordinate, and intensive sampling in these windows is achieved by enforcing an external biasing potential. The samplings in each window must overlap with adjacent windows, so that the unbiased PMF can be reproduced by removing the biasing potential. The external biasing potential *u*_*i*_ at window *i* is a harmonic function *u*_*i*_ *= k*_*i*_(*r* – *r*_*i*_ *)* ^2^, where *r*_*i*_ is the reference position, and *k*_*i*_ is the harmonic force constant. The force constant *k*_i_ was typically set to 1000 kJ·mol^–1^·nm^–2^, but elevated in cases where inadequate conformational sampling within the window was observed. We used WHAM^146^ to remove the biasing potential and reconstruct the PMF.

### Data Analysis

The VMD^147^ and PyMOL^38^ software were adopted to visualize the trajectories and configurations of the MD simulations. The calculation of the root-mean-square-deviation (RMSD), root-mean-square-fluctuation (RMSF), distance, and solvent-accessible surface area (SASA) was finished using MDAnalysis.^148^

If any atom of a residue came within 4.5 Å of any Au atom in a given frame, we defined that residue as being in contact. To mitigate interference from noncontact frames and facilitate cross-simulation averaging, we introduced a contact ratio for each residue: the fraction of frames where the individual residue contacts the AuNP surface, given that the protein is already in overall contact with the AuNP (there is at least one residue in contact). The contact residues in the VEGF_165_/AuNP complex were calculated using MDanalysis.^148^

Hydrogen bond analysis was conducted using VMD.^147^ A hydrogen bond is formed between an atom with a hydrogen bonded to it (the donor, D) and another atom (the acceptor, A) provided that the distance D–A is less than the cutoff distance (3.0 Å) and the angle D-H-A is less than the cutoff angle (20 degrees).

All graphs from the 2000 ns simulations employing a moving average were smoothed using a window size of 200 ns, while those from the 10 ns US simulations used a window size of 1 ns.

For the hydrogen bond percentage distribution (Figure 1F), error bars were calculated as the standard deviation of 1000 bootstrap resamples of the pooled H-bond count data across all four replicates.

## Supplementary Material



## Data Availability

The input files for this study are available on Zenodo at DOI: 10.5281/zenodo.17641714. Coordinate files were obtained from AlphaFold server as described in Methods. The software used in this study is as follows: GROMACS (v2023.2), VMD (v1.9.4), Pymol (v3.1.1), and MDAnalysis (v2.8.0).

## Notes

Additional figures showing PCA results, H-bond analysis, and snapshots of key interactions during MD simulations (Figures S1–S3); initial orientations and contact analysis of VEGF165 with AuNPs (Figures S4 and S5); structural comparison of 5A mutant and wild-type VEGF165 (Figure S6); identification of mutated VEGF165 and TEM characterization of AuNPs (Figures S7 and S8); SMD force profiles and distance analysis for binding interactions (Figures S9 and S10); convergence analysis of PMF calculations (Figure S11); cytotoxicity analysis (Figure S12); quantitative structural metrics of VEGF_165_ during the extended and compact phases (Tables S1); treatment media composition (Tables S2); original ELISA data (Tables S3) (PDF)
