# Supplementary material for: Inhibition of VEGF165-Induced Angiogenesis by Gold Nanoparticles through HQGQH as the Primary Binding Site
Source: J Chem Inf Model. 2026 Apr 17;66(9):5519–32. doi: 10.1021/acs.jcim.5c02810 (PMC13169350; doi:10.1021/acs.jcim.5c02810)
Supplement: Supplementary file 1 [file ci5c02810_si_001.pdf]

Inhibition of $VEGF_{165}$-Induced Angiogenesis by Gold Nanoparticles through HQGQH as the Primary Binding Site

Jiayi Wan[1, a], Yihan Zhou[1, a], Jiajun Zhu[1, a], Yutong Lu[a], Yuting Lyu[a], Hongke Hao[a], Zhiliang Lu[a], Carrie A. Duckworth[b], Yiheng Liu[a], Hanyu Zhou[a], Yulan Yi[a], Kevin Chun Chan[*, a] and Xia Huang[*, a]

[1] These authors contributed equally to this work.

[a] Department of Biosciences and Bioinformatics, Xi'an Jiaotong-Liverpool University, Suzhou, Jiangsu 215123, PR China

[b] Institute of Systems, Molecular and Integrative Biology, The University of Liverpool, Biosciences Building, Crown Street, L69 7BE, United Kingdom

*Corresponding author:

Xia Huang: Xia.Huang@xjtlu.edu.cn

*Co-corresponding author:

Kevin Chun Chan: Chun.Chan@xjtlu.edu.cn

Table of Contents

Supporting Figure S1. PCA of four independent $VEGF_{165}$ simulation trajectories.

Supporting Figure S2. Number of H-bonds between dangling regions.

Supporting Figure S3. Another two key H-bonds of $VEGF_{165}$ formed during the conformational change.

Supporting Figure S4. Representative initial orientations of $VEGF_{165}$ relative to AuNP.

Supporting Figure S5. Other residues with high contact ratio.

Supporting Figure S6. Structural analysis of the 5A mutant $VEGF_{165}$ compared with WT $VEGF_{165}$.

Supporting Figure S7. The detection of mutated $VEGF_{165}$.

Supporting Figure S8. TEM for AuNPs detection.

Supporting Figure S9. SMD force profile with multi-region binding.

Supporting Figure S10. SMD analysis for HQGQH-only binding.

Supporting Figure S11. Convergence analysis of PMF calculations.

Supporting Figure S12. The cytotoxicity AuNPs on endothelial cells.

Supporting Table S1. Structural metrics of the $VEGF_{165}$ for the 0-750 ns phase and the 750-2000 ns phase.

Supporting Table S2. Treatment medium for hCMEC/D3 cells.

Supporting Table S3. The original data of ELISA.

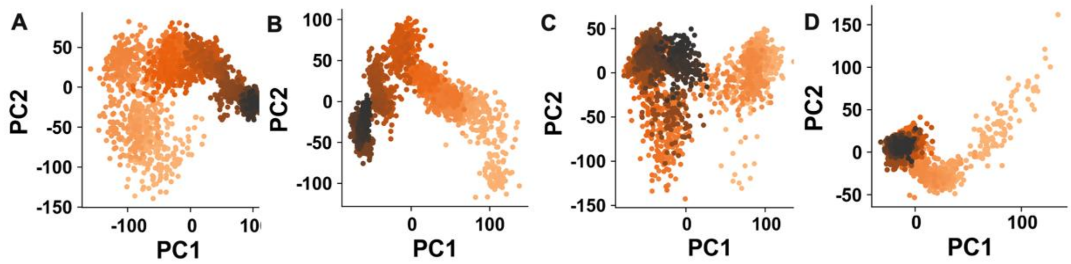

Supporting Figure S1. PCA of four independent VEGF$_{165}$ simulation trajectories. A-D. PCA results based on atomic displacements relative to the reference frame, with instantaneous conformations colored from dark to light in order of time.

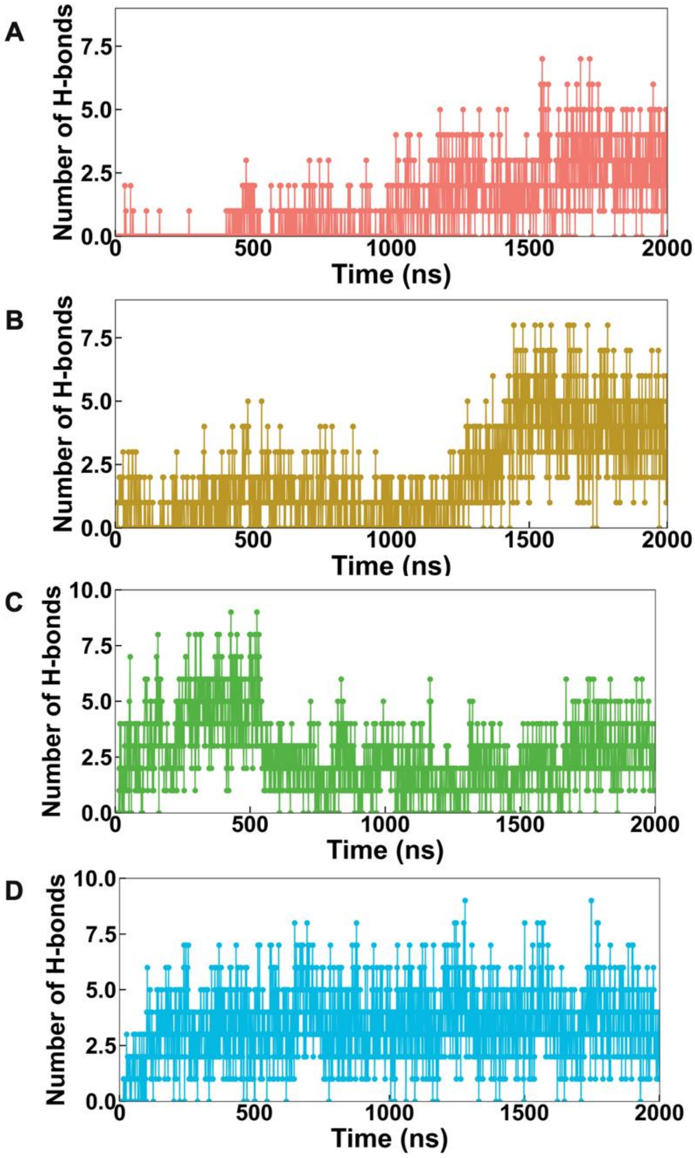

Supporting Figure S2. Number of H-bonds between dangling regions. A-D. Number of H-bonds formed between the two dangling regions during four independent MD simulations of $VEGF_{165}$, indicating a similar increased trend over time.

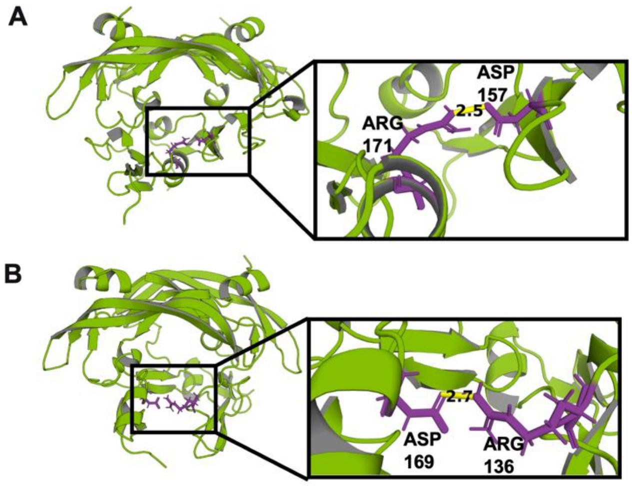

Supporting Figure S3. Another two key H-bonds of VEGF$_{165}$ formed during the conformational change. A. Snapshot of the VEGF$_{165}$ showing the H-bond between ARG171 and ASP157 with a distance of 2.5 Å. B. Snapshot of the VEGF$_{165}$ showing the H-bond between LYS169 and GLU136 with a distance of 2.8 Å.

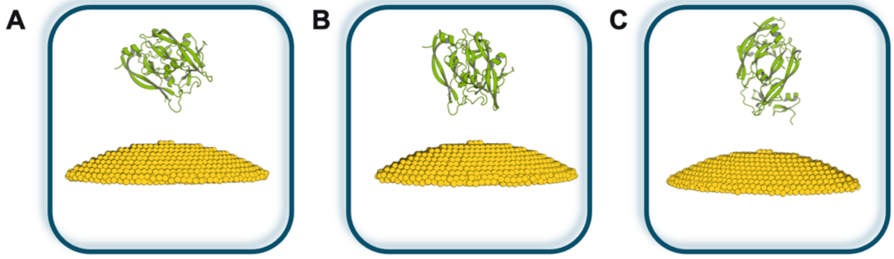

Supporting Figure S4. Representative initial orientations of VEGF$_{165}$ relative to AuNP. A-C. Three representative starting structures selected from the 21 distinct conFigureurations.

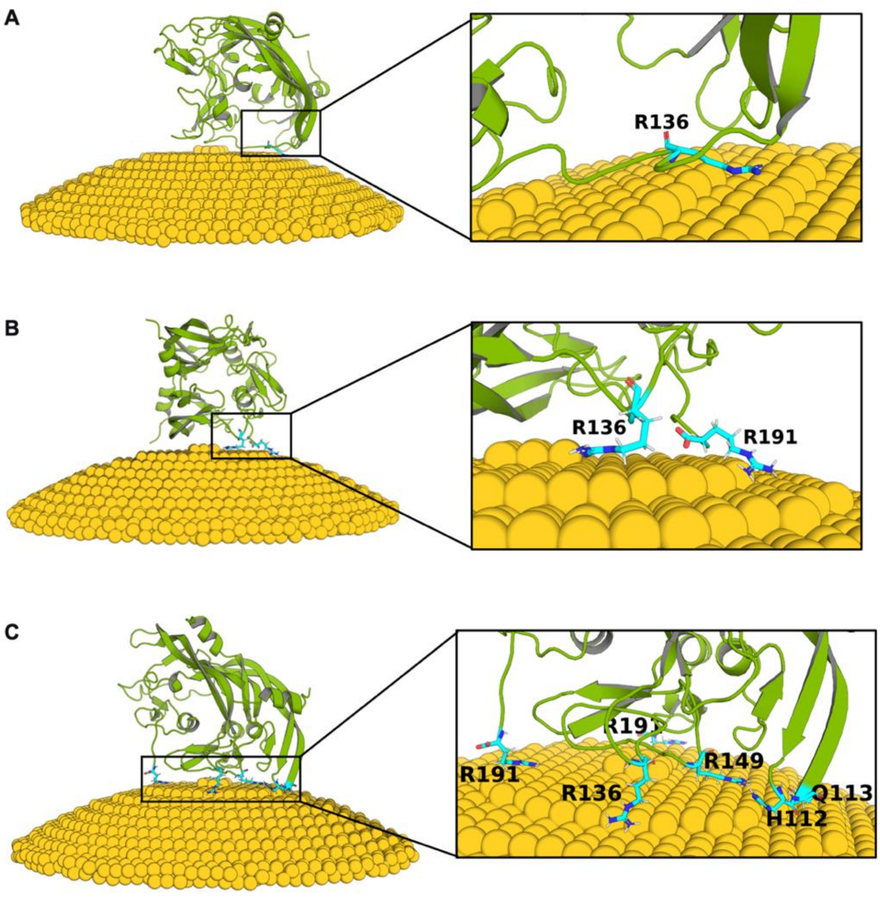

Supporting Figure S5. Other residues with high contact ratio. A. 136R contacts with AuNP. B. 136R and 191R contact with AuNP. C. 136R, 149R and 191R contact with AuNP.

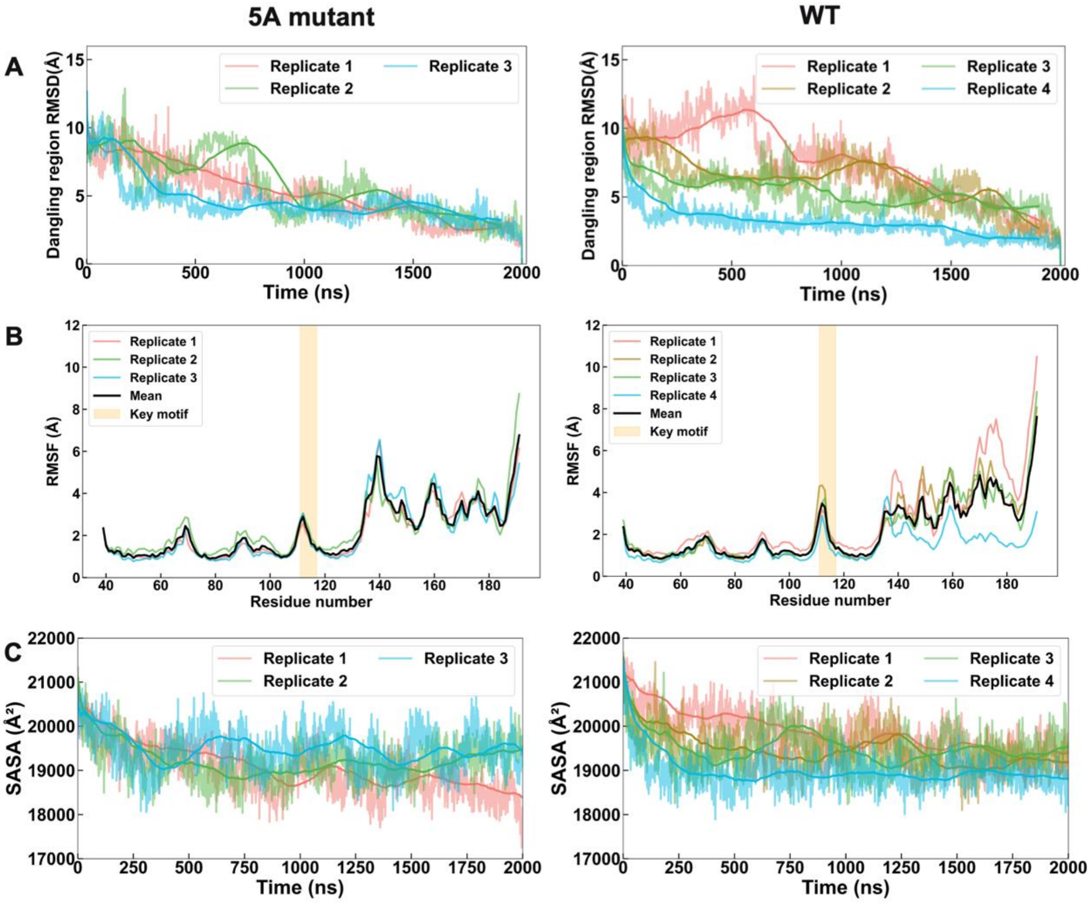

Supporting Figure S6. Structural analysis of the 5A mutant VEGF$_{165}$ compared with WT VEGF$_{165}$. A. RMSD of the 5A mutant (left) and WT (right) VEGF$_{165}$ dangling regions, aligned to the final frame of VEGF$_{165}$ from four 2000 ns MD simulation replicates. B. RMSF of the 5A mutant (left) and WT (right) VEGF$_{165}$. C. SASA of the entire protein over time. In A and C, raw data are shown as translucent lines and running averages are shown as bold lines.

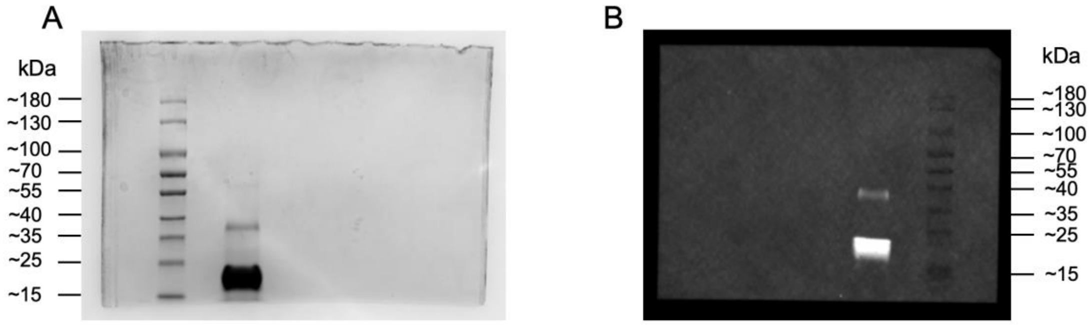

Supporting Figure S7. The detection of the mutated $VEGF_{165}$. A. Coomassie blue staining was used to identify the constructed mutated $VEGF_{165}$ (19.9 kDa). B. Western blot was used for further identification.

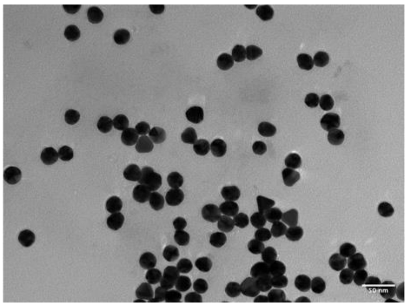

Supporting Figure S8. TEM for AuNPs detection. The diameter of AuNPs was around 20 nm with a spherical morphology.

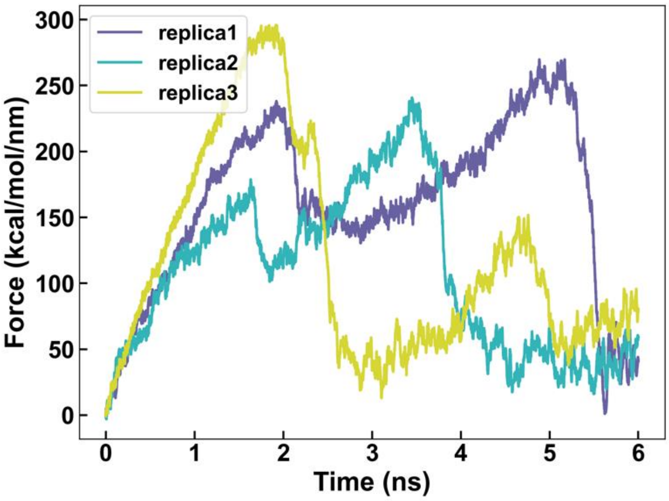

Supporting Figure S9. SMD force profile with multi-region binding. Pulling force profile during SMD in a WT–AuNP system where the protein attached via multiple, non-specific sites in addition to the primary HQGQH motif.

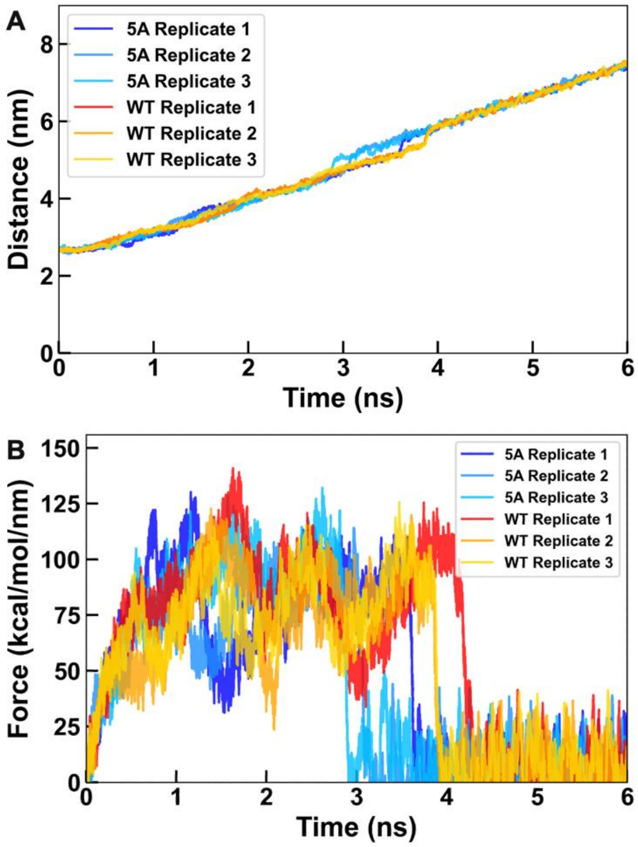

Supporting Figure S10. SMD analysis for HQGQH-only binding. A. The COM distance between the core region of WT/5A mutant and AuNP over time. B. The corresponding force profile required to pull the protein away from the nanoparticle surface.

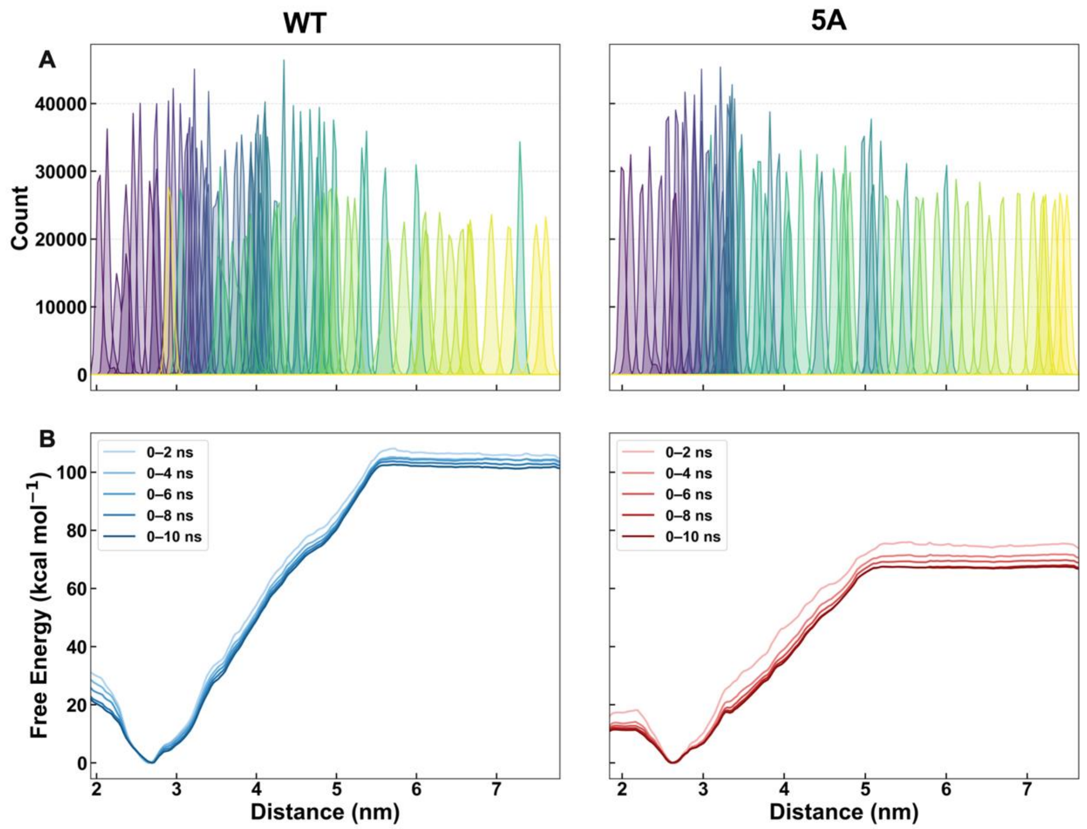

Supporting Figure S11. Convergence analysis of PMF calculations. A. Histograms of the sampling windows for the WT (left) and 5A mutant (right). B. Time-dependent convergence of the PMF profiles for the WT (left) and 5A mutant (right). The PMF curves were calculated using WHAM over progressive simulation time intervals (0–2 ns, 0–4 ns, 0–6 ns, 0–8 ns, and 0–10 ns).

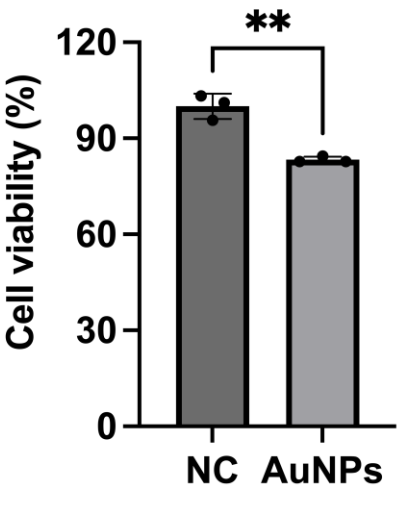

Supporting Figure S12. The cytotoxicity AuNPs on endothelial cells. The cytotoxicity of 5 μM AuNPs on hCMEC/D3 cells following a 24 h exposure was assessed using the CCK8 assay. AuNPs decreased cell viability to 83%±1%, showing low cytotoxicity. Data showed as mean ± SD. Statistical analysis was performed by two-tailed unpaired Student's t-test. NC: negative control.

Supporting Table S1. Mean and standard deviation (Std) of structural metrics of the $VEGF_{165}$ for the 0-750 ns phase and the 750-2000 ns phase.

Metric	Phase	Mean	Std
Dangling RMSD	0–750 ns	6.7	2.5
Dangling RMSD	750–2000 ns	4.5	1.9
Dangling Distance	0–750 ns	22.3	3.5
Dangling Distance	750–2000 ns	20.3	2.1
Dangling–Rest Contacts	0–750 ns	1560	340
Dangling–Rest Contacts	750–2000 ns	1760	350
Two-Dangling Contacts	0–750 ns	430	320
Two-Dangling Contacts	750–2000 ns	620	300
Protein SASA	0–750 ns	19610	640
Protein SASA	750–2000 ns	19280	430

The 0–750 ns and 750–2000 ns time intervals approximate the extended and compact conformational states of $VEGF_{165}$, respectively. RMSD and Distance are measured in Å, and SASA is measured in $Å^2$.

Supporting Table S2. Treatment medium for hCMEC/D3 cells.

Group	Treatment medium
Negative control (NC)	complete endothelial cell medium
Wild-type $VEGF_{165}$ (WT)	40 ng/ml WT in complete endothelial cell medium
WT+AuNPs	40 ng/ml WT and 5 μM AuNPs in complete endothelial cell medium
5A mutated $VEGF_{165}$ (5A mutant)	40 ng/ml 5A mutant in complete endothelial cell medium
5A mutant+AuNPs	40 ng/ml 5A mutant and 5 μM AuNPs in complete endothelial cell medium

The endothelial cell treatment medium was used to treat hCMEC/D3 cells in different groups. WT or 5A mutant with or without AuNPs was dissolved in complete endothelial cell medium.

Supporting Table S3. The original data of ELISA.

	WT (Total $VEGF_{165}$)	WT+AuNPs (Unbound $VEGF_{165}$)	5A mutant (Total $VEGF_{165}$)	5A mutant+AuNPs (Unbound $VEGF_{165}$)
Replicate 1	0.298	0.205	0.095	0.090
Replicate 2	0.254	0.218	0.094	0.087
Replicate 3	0.286	0.203	0.083	0.089

The original data of binding rate measurements by ELISA. Totally, three replicates were

performed. The proteins bound to AuNPs were calculated by subtracting the unbound proteins from the total proteins.
